# Intrinsic protein disorder in histone lysine methylation

**DOI:** 10.1186/s13062-016-0129-2

**Published:** 2016-06-30

**Authors:** Tamas Lazar, Eva Schad, Beata Szabo, Tamas Horvath, Attila Meszaros, Peter Tompa, Agnes Tantos

**Affiliations:** Institute of Enzymology, Research Centre for Natural Sciences, Hungarian Academy of Sciences, Magyar tudósok körútja 2, 1117 Budapest, Hungary; Pázmány Péter Catholic University, Faculty of Information Technology and Bionics, Práter utca 50/a, 1083 Budapest, Hungary; VIB Structural Biology Research Center (SBRC), Pleinlaan 2, 1050 Brussels, Belgium; Vrije Universiteit Brussel, Pleinlaan 2, 1050 Brussels, Belgium

**Keywords:** Histone lysine methyltransferases, Posttranslational modifications, MLL proteins, Intrinsically disordered protein regions, Linear motifs, Binding regions, Epigenetics

## Abstract

Histone lysine methyltransferases (HKMTs), catalyze mono-, di- and trimethylation of lysine residues, resulting in a regulatory pattern that controls gene expression. Their involvement in many different cellular processes and diseases makes HKMTs an intensively studied protein group, but scientific interest so far has been concentrated mostly on their catalytic domains. In this work we set out to analyze the structural heterogeneity of human HKMTs and found that many contain long intrinsically disordered regions (IDRs) that are conserved through vertebrate species. Our predictions show that these IDRs contain several linear motifs and conserved putative binding sites that harbor cancer-related SNPs. Although there are only limited data available in the literature, some of the predicted binding regions overlap with interacting segments identified experimentally. The importance of a disordered binding site is illustrated through the example of the ternary complex between MLL1, menin and LEDGF/p75. Our suggestion is that intrinsic protein disorder plays an as yet unrecognized role in epigenetic regulation, which needs to be further elucidated through structural and functional studies aimed specifically at the disordered regions of HKMTs.

**Reviewers:** This article was reviewed by Arne Elofsson and Piotr Zielenkiewicz.

**Electronic supplementary material:**

The online version of this article (doi:10.1186/s13062-016-0129-2) contains supplementary material, which is available to authorized users.

## Findings

The complex pattern of posttranslational modifications (PTMs) of histone proteins result in an epigenetic regulatory code that controls entire gene expression programs within a cell [1]. One of the best characterized histone modifications is methylation, that can occur on lysine or arginine residues [2]. Lysine methylation is mediated by histone lysine methyltransferases (HKMTs), a protein family defined by the presence of the SET domain, named after the Drosophila proteins Suppressor of variegation 3–9 [Su(var)3–9], Enhancer of zeste [E(z)], and Trithorax (Trx) [2]. DOT1L is the only protein that is capable of lysine methylation, despite the absence of a SET domain [3]. Many HKMTs are involved in different types of cancer [4, 5], making them an intensively studied protein group. While most studies are directed to the catalytic domain(s), we aimed at the structural analysis of the regions of HKMTs outside their catalytic domains. After finding that a significant proportion of the studied sequences are predicted to be intrinsically disordered, we tried to identify possible functional sites within these regions.

Intrinsically disordered proteins and protein regions (IDPs/IDRs) lack stable 3D structure in their functional state that confers a multitude of functional advantages [6], utilized in the diverse roles of IDPs in important biological processes [7, 8].

Although proteins participating in chromatin remodelling are known to have high levels of disorder in general [9], HKMTs distinguish themselves from other histone modifying enzymes not only by a high level of disorder (Additional file 1: Figure S1A), but also by the length of their disordered regions. Whereas 60 % of HKMTs contain IDRs longer than 80 amino acids (Additional file 1: Figure S1B), less than 20 % of eukaryotic proteins contain IDRs of similar length [10].

An evolutionary comparative analysis shows that the average length and number of proteins responsible for histone modification increases with evolution, with the sharpest transitions occurring between prokaryotes and eukaryotes and lower eukaryotes and vertebrates. Although the statistical analysis is hindered by large standard deviation values and the limited sample size in certain cases (for example, the histone demethylase group of protostomes contains only one representative and there are only two known bacterial histone arginine methyltransferases), HKMTs are almost always significantly longer than other histone modifying enzymes (Additional file 2: Figure S2). This is not true for prokaryotes, where HKMTs constitute the shortest proteins of the studied groups. This observation also includes that the length of HKMTs rose more sharply in eukaryotes than in other histone modifying enzyme families. Since it was shown earlier that protein length and intrinsic protein disorder does not correlate closely with organism complexity [11], this elevated length is probably related to the specific function and regulation of the HKMT proteins rather than being a general evolutionary trait. Contrary to protein length, the number of HKMT proteins is generally not significantly higher than other histone modifying enzymes in the studied evolutionary groups, although in some specific cases we could detect significant differences. This finding shows that in case of HKMTs, more complex regulation with growing organism complexity was achieved through extending individual proteins, rather than producing more, specialized representatives of the family.

In order to determine the evolutionary variability of these IDRs, we performed disorder conservation analysis of the mostly disordered HKMTs, using the DisCons [12] webtool, which can differentiate between constrained and flexible disorder. Disorder is considered constrained when disorder tendency and sequence of a region are both conserved, while flexible disorder means that only the disorder tendency is retained through evolution. The analysis showed that the long IDRs are highly conserved in vertebrates, with constrained disorder conservation levels above 80 % for all examples (Additional file 3: Table S1). Since disordered proteins generally tolerate sequence changes better than globular proteins [13, 14], the fact that not only disorder, but also sequences are conserved, shows that these regions harbor important functional sites. CREB-binding protein (CBP), a histone acetyltransferase with experimentally confirmed functions in its disordered regions [15], has a similar disorder conservation level as the studied HKMTs.

Although many HKMTs contain single amino acid repeat regions (see Additional file 4: Table S2.) and other low complexity regions (LCRs), SEG analysis [16] shows that contrary to protein disorder, LCRs are not overrepresented in any of the histone modifying protein families compared to the average of human proteins, and the overlap between LCRs and IDRs is limited (Additional file 1: Figure S1C.). This suggests that although LCRs are thought to be involved in mediating flexible protein-protein interactions [17], it seems that in this particular case, low complexity is not a dominant feature.

Polyglutamine sequences are among the most studied LCRs due to their involvement in many diseases [18]. Of HKMTs, only MLL4 contains long stretches of polyglutamine (polyQ) repeats (14 regions with lengths between 5 and 13 amino acids). A long run of glutamines between aa 3898 and 3974 is also found in MLL4, where Q repeats are interrupted with a leucine residue at every five to ten residues. This region is predicted to form a coiled-coil structure [19] and may be involved in stabilizing protein-protein interactions as suggested for such regions by Schaefer et al. [20]. It is to be noted that LCRs also often have highly repetitive Q/N-rich regions, which may undergo regulated structural transitions from a disordered to a highly ordered amyloid-like state, conferring prion-like functions on the protein [21].

The main functional regions of IDPs/IDRs, however, are short recognition elements, most often termed eukaryotic linear motifs (ELMs). A search in the ELM database [22] for known sequence motifs in the disordered regions of HKMTs resulted in a limited number of annotated motifs, but we could identify more ELM hits with the database’s acceptable expectation value. One of the most frequent motifs found was LIG_WD40_WDR5_WIN_1 which is responsible for binding WDR5 and WD40 domains [23].

Other motifs with reliable e-values are involved in transcriptional activation/repression, cellular proliferation, ubiquitination, DNA repair, RNA binding and splicing. These are in good correlation with the functions generally assigned to HKMTs [2], but the physiological role of these predicted motifs remains to be experimentally validated. We found 18 different motifs altogether, and these occurred at 50 different sites in the mostly disordered HKMTs (Additional file 4: Table S2). This represents more than 2 predicted ELM motifs per 1000 residues, which is significantly higher than the number obtained for randomized sequences with the same amino acid composition (0.645 ELM motif per 1000 residues, *p* < 0.0001). The average level of conservation of motifs predicted in MLL1 and MLL4 is significantly higher (*p* = 0.001 and *p* = 0.003, respectively) than that of the whole proteins, but ELM motifs in the other, highly disordered HKMTs (NSD1, SUV420H1, PRDM2 and DOT1L) do not show significantly higher conservation. Given that the average conservation of these proteins is already rather high, this does not necessarily question their functional importance. Our suggestion is that some, or many of the ELMs found in this study may participate in the interactions of HKMTs with other macromolecules, making them excellent candidates for further investigations. A statistical analysis shows that ELMs participating in protein-protein interactions occur at a significantly (*p* < 0.0001) higher level in the studied HKMTs (1.7814 motifs/1000 aa) than in randomized sequences (0.995 motifs/1000 aa), underscores this proposition. It is also informative that research directed at the non-enzymatic regions of HKMTs has already unveiled a new motif that mediates the interaction of different proteins with LEDGF/p75 [24].

**Fig. 1:**
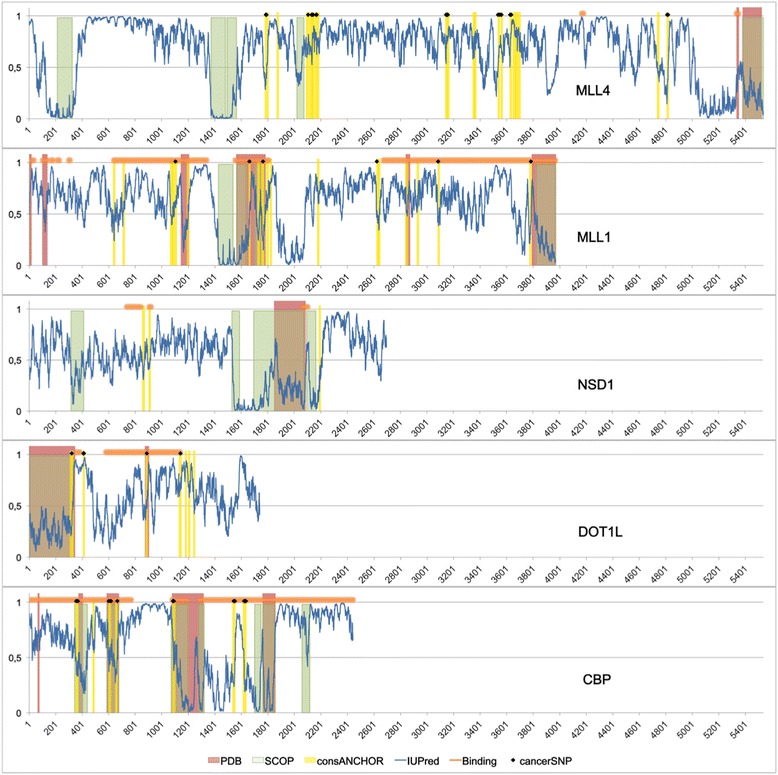
IUPred profile of four representative HKMTs and CBP. Regions with corresponding PDB structures (red^1^), SCOP domains (green), conserved predicted binding regions (yellow), known binding regions (orange horizontal lines) and cancer associated SNPs (black diamonds) are indicated. ^1^List of PDB structures: MLL1: 4gq6_b, 3u88_m, 2mtn_a, 2msr_a, 2j2s_a, 2kyu_a, 3lqh_a, 2agh_c, 2w5y_a; MLL4: 3uvk_b, 3erq_d, 4z4p_a; NSD1: 3ooi_a; DOT1L: 3uvp_a, 2mv7_b; CBP: 1rdt_e, 1lik_a, 2lxt_a, 4n4f_a, 2kje_a

Using the ANCHOR [25] server that can predict disordered binding regions from sequence, we mapped the potential binding regions of the IDRs of HKMTs. The number of ANCHOR sites (30.106 regions/1000 aa) is significantly higher (*p* < 0.0001) than that found in the randomized sequences (25.935 regions/1000 aa). In order to reduce the number of false positive hits, we only considered the ANCHOR sites that were conserved in vertebrates (Fig. 1). A comparison with different databases containing cancer-related mutations resulted in several hits localized to these putative binding regions. Since HKMTs work mainly as parts of large complexes [2], it is not unfounded to suggest that these may be the regions responsible for mediating functionally important interactions. A recently characterized DOT1L-AF9 interaction [26] overlaps with a predicted binding site (Fig. 1), pointing to the validity of our suggestions. AF9 is a fusion partner of MLL1 and is involved in the leukemias involving MLL fusions [27], which highlights the importance of DOT1L-AF9 interaction.

The disordered region of DOT1L is probably also involved in the H2BUb-H3K79 crosstalk, since a C-terminal truncated construct can methylate nucleosomes in the absence of the facilitating ubiquitin (Ub) mark [28]. Ubiquitin interaction appears to be mediated through lysine-rich regions in DOT1 proteins, as shown for yeast DOT1P [29]. The lysine-rich region of yeast DOT1P localizes in the disordered region of the protein and human DOT1Ll also contains a disordered lysine-rich region that might be involved in the H2BUb-H3K79 crosstalk. The lysine-rich region homologous to that of yeast DOT1P is localized between amino acids 387–416 in DOT1L, and overlaps with a conserved ANCHOR site (aa 408–416) according to our prediction (Fig. 1). The notion that it is a valid and important interaction site is corroborated by the three SNPs in this region that are found in cancer databases.

NSD1 also uses a disordered region for interacting with Nizp1 in mediating gene repression [30]. The interacting region of mouse NSD1 is a cysteine-rich region (aa 2117–2207) that corresponds to a conserved ANCHOR sequence in the human protein (Fig. 1), raising the possibility of a similar mechanism in human cells.

MLL proteins also contain several conserved ANCHOR regions, some of them in longer sequences that are known to participate in structurally not characterized partner binding (Fig. 1). The reliability of our predictions is supported by the finding that the ternary complex formed between the activation domain of MLL1, the KIX domain of CBP and the TAD of c-Myb [31] is mediated by a short sequence in MLL1 between residues 2844 and 2857 [32]. This interaction is essential for transcriptional activation by MLL [31] and overlaps with one of the conserved ANCHOR sites (aa 2841–2853).

The functional importance of regions of MLL proteins other than their SET domain is underlined by the fact that unlike *mll*^*−/−*^ mice, animals with SET domain-deleted MLL are viable and fertile, although they show defects in DNA methylation [33]. The SET domain is also lost in MLL rearranged leukemias, where the N-terminal region of MLL proteins is fused to various protein partners, resulting in aberrant expression of MLL target genes [34]. The disordered nature of the MLL protein is important for the fusion proteins to be viable in the cells, as a link between protein disorder and fusion protein survival was shown in a previous work [35].

The extreme length of the IDRs found in HKMTs suggests that these regions have further roles than simply presenting interaction sites of a couple of amino acids in length. Involvement of long disordered regions in establishing long-range contacts between spatially distant binding partners was suggested for proteins participating in nonsense-mediated decay [36]. HKMTs might rely on similar strategies when recognizing other histone modifications, exemplified by the H2BUb-H3K79 crosstalk in the case of DOT1L. These long IDRs may also serve as tools for complex intramolecular regulation through the interplay of a variety of elements, domains, motifs and linkers in a phenomenon termed ‘multistery’ [37].

Although disordered regions do not fold into a well-defined structure on their own, they often gain structure upon binding to different partners through induced folding [38]. The ternary complex formed between MLL1, menin and LEDGF/p75, critical for the development of MLL leukemia [39], is a good example of a well-characterized interaction involving disordered regions. We demonstrate how a disordered segment can change the stability of a complex through the analysis of the published structures supplemented with molecular modeling.

**Fig. 2:**
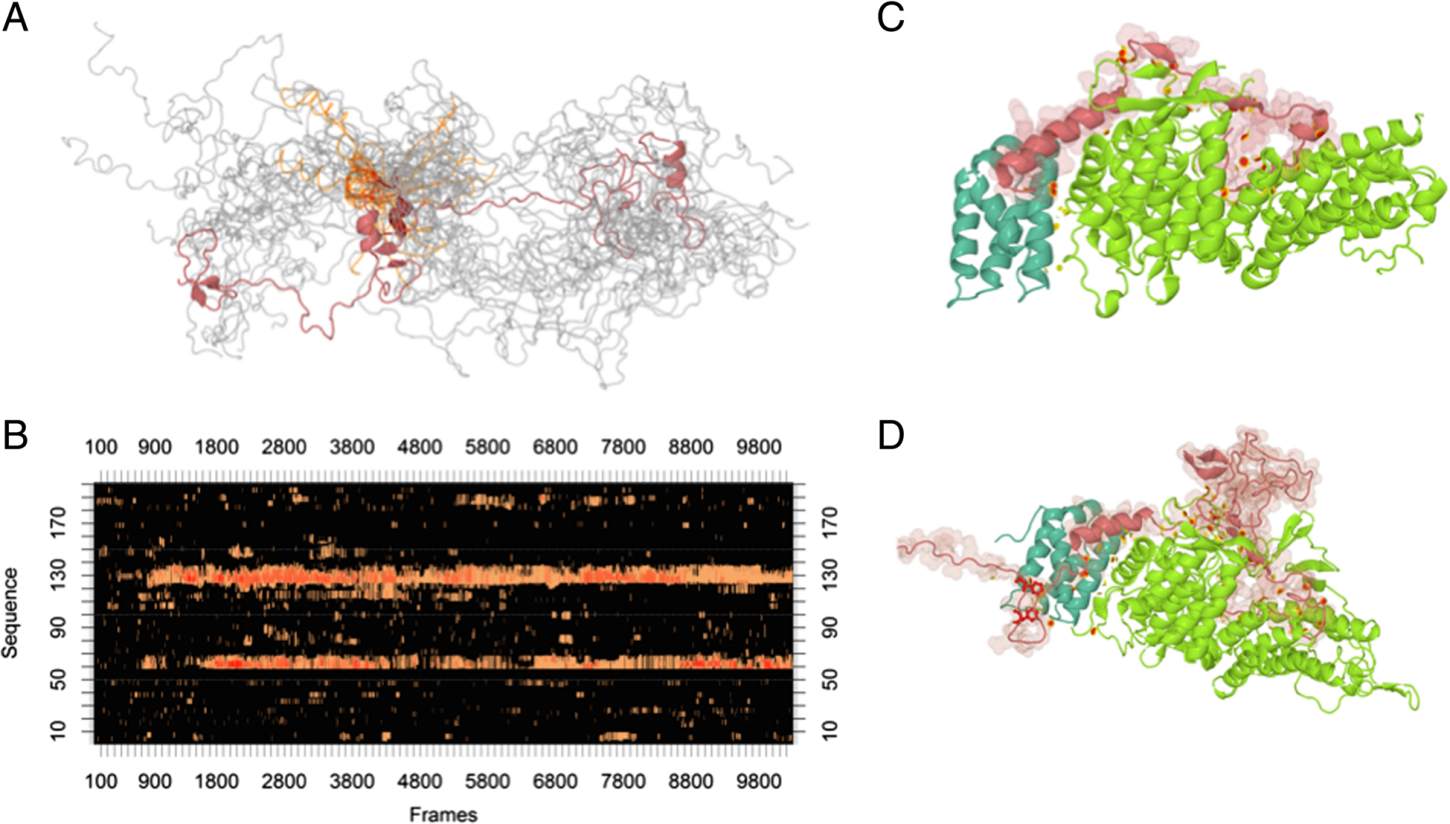
Molecular dynamics simulation of MLL1-menin-LEDGF/p75 complex. **a** Overlay of 20 structures between 900 k-1100 k steps of DMD of free MLL1 N-terminus. The region between amino acids 120–135 are highlighted in red or orange. **b** DSSP helix content of the free MLL1 N-terminus per frame versus the amino acid chain. Orange to red lines represent the number of replicas (one, two or three) in any given frame that contain an amino acid in helical conformation (1–2000 k steps). **c** Structure of the ternary complex as represented in PDB database (3U88). Salmon: MLL1, cyan: LEDGF/p75, green: menin. Side chains of F148 and F151 in MLL1 are red. Intramolecular contacts are shown as yellow-red dots. **d** Structure of the ternary complex as modeled with the disordered regions of MLL1 based on PDB structures 3U88 and 2MSR. Salmon: MLL1, cyan: LEDGF/p75, green: menin. Side chains of F148 and F151 in MLL1 are red. Intramolecular contacts are shown as yellow-red dots (at 200 k steps)

The originally published crystal structure (PDB: 3U88) contained a region spanning amino acids 4 to 153 of MLL1 from which the disordered segments (aa 16–22 and 36–102) were removed [40]. We performed molecular dynamics simulations using the sequence of MLL1 between amino acids 1 and 200. Our simulations show that this region is highly dynamic in the unbound state, sampling a multitude of different conformations (Fig. 2a), with short regions of limited preference for secondary structural elements. The region between amino acids 120–140 has the highest tendency to fold into a continuous alpha helical state which is capable of facilitating binding (Fig. 2b). The ensuing conformational selection is a basic mechanism of disordered proteins binding to their binding partners [38].

A short segment of MLL1 (aa 140–160) binds LEDGF/p75 independently of the formerly described helix [41, 42] through a region that does not fold upon binding and has no particular structural propensity in the unbound state (PDB: 2MSR, Fig. 2b). This is an example of the unique ability of IDPs to bind without folding [43], which is nevertheless very important for the stability of the ternary complex. Our molecular dynamics simulations demonstrate that the LEDGF/p75-menin complex is not stable, (Additional file 5: Movie 1) and while the MLL1 helix between menin and LEDGF/p75 stabilizes the ternary complex with hydrophobic and electrostatic interactions (Additional file 6: Movie 2), the extensive movements of LEDGF/p75 relative to menin might not be compatible with the biological function. Even though the two interacting amino acids of the disordered loop (F148 and F151) do not form stable bonds with the partners, the simulation containing the loop region showed a much more stable complex (Additional file 7: Movie 3). The MLL1 construct in the published crystal structure also contains the binding phenylalanines, but no coordinates could be assigned to them [40], revealing that they remain disordered even in the confines of a crystal lattice. This model illustrates nicely that two different IDR binding strategies (folding upon binding and binding without folding) can work together to modulate the stability of a binding interface. The fact that in the case of the MLL_6–153_ there could be no interaction detected with LEDGF/p75 [40] but the region 1–160 interacts with LEDGF/p75 alone [41, 42], hints at the importance of amino acids distant to the actual binding site. This observation underlines that even though many IDP interactions are mediated by residual structural elements, lack of a tendency to fold does not necessarily mean a lack of interaction capability and function.

Apart from the known binding regions of MLL1, our simulations also included a large disordered loop of MLL1 between amino acids 36 and 102. The larger number of intramolecular contacts in the model compared with the crystallized complex (68 versus 45, respectively) suggests that disordered regions distant to the binding site may also contribute to the interaction (Fig. 2c and d). The loop region does not seem to make extensive contacts with either partners and might serve as a platform for other interaction partners.

In all, we have shown that intrinsic disorder is a prominent feature of HKMTs and the intricate regulation and complex activity of these important enzymes cannot be fully understood without dissecting the behavior of these regions. The rare instances where disordered regions of HKMTs were studied show that many important functions lie in these sequences. Given the extreme length of IDRs in some of the HKMTs, it is entirely possible that many other functions await discovery. For this reason it is important to direct structural and biochemical studies at the disordered segments of these proteins. Most promising candidates would be the conserved ANCHOR regions, especially those that contain cancer-related SNPs. Regions participating in detected, but uncharacterized partner binding also bear the possibility of notable discoveries. Recognizing the importance of protein disorder in the epigenetic regulation is important for a deeper understanding that may bring further development of this field.

## Methods

The human histone lysine methyltransferase (HKMT) dataset was taken from an article published in 2013 about the SET domain containing histone methyltransferases [2]. DOT1L was added manually as the only HKMT lacking a SET domain. UniProt Acc-s and information about the length of the protein sequences were collected from the UniProt database.

Histone modifying enzymes were collected from the UniProt database by searching with the enzyme names and the appropriate GO annotations: ‘histone-lysine N-methylase activity’, ‘histone-arginine N-methylase activity’, ‘histone acetyltransferase activity’, ‘histone demethylase activity’ and ‘histone deacetylase activity’. Protein existence was ‘not uncertain’ and fragment sequences were left out. Of the different variants of the same protein, only the longest version was used for analysis. The human dataset consisted of 34 HKMTs, 8 HRMTs, 29 HATs, 22 Histone demethylases and 18 Histone deacetylases. The datasets used for evolutionary analysis contained 2230 HKMTs, 374 HRMTs, 4444 HATs, 539 Histone demethylases and 2038 Histone deacetylases. Evolutionary groups were formed and named as follows: Bacteria, Archea, Eukaryotic unicellular (Eukaryotic species that are not plants, metazoans or fungi), Fungi, Metazoa1 (Metazoans except for protostomes and deuterostomes, e.g. sponges, cnidarians), Metazoa2 (Protostomes except for Ecdysozoa, e.g. flatworms, annelids, molluscs), Metazoa3 (Ecdysozoa), Metazoa4 (Deuterostomes except for vertebrates), Metazoa5 (Vertebrates). For this analysis reference proteomes were used only.

Structural disorder was predicted with the IUPred long disorder predictor [44]. The overall disorder rate was computed as the fraction of residues with an IUPred score of at least 0.5. To evaluate the IUPred long disorder prediction, we compared its scores to the results given by nine other disorder predictions from MobiDB [45]. IUPred gives the same per aa classification as the consensus in >90 % of the sequences (Average IUPred disagreement: 9.4 ± 4.7 % st.error).

We searched human linear motifs in the disordered HKMTs (disorder rate higher than 50 %) in the ELM database [22], and only collected the ones annotated from the literature and the hits with e-value < 0.0001, both with nuclear localization. The construction of scrambled sequences to check for the significance of the frequency of ELM hits in HKMTs was made by shuffling the amino acid residues of the above mentioned HKMTs having an IUPred score at least 0.5, using a Perl script. Twenty constructs were generated with length of 10000 residues for a 10x sequence coverage.

PDB structures were searched manually, while SCOP domains were assigned with the help of annotations in the D2P2 database [46]. Literature mining for known binding regions of HKMTs was done by reading the evidence references of the interaction hits found in the BioGrid database [47]. Cancer-related single nucleotide polymorphisms in the long conserved IDR regions were collected from the BioMuta v2.0 [48] and COSMIC databases [49]. Long conserved disordered binding regions were calculated in two steps: first, we predicted longer (min. 8 residues) disordered binding regions by ANCHOR [25]. Next, we took the intersection of the set of these regions with the Scorecons [50] conservation output (with default *valdar01 scoring*) defining “constrained” regions with a value of at least 0.9, based on a multiple alignment generated out of 22–24 vertebrate orthologs. The multiple sequence alignment was generated in UniProt selecting the “canonical” sequences from the vertebrate organisms, ignoring fragments, using BLAST with default parameters (Clustal-Omega alignment, Gonnet transition matrix, gap opening penalty 6 bits, gap extension 1 bit). Each vertebrata multiple alignment file of the proteins contained a broad range of species from primates to the earliest diverged fishes.

The calculation of sequence conservation and disorder conservation was carried out by DisCons [12], from alignments with default parameters (IUPred long, Jensen-Shannon divergence, window size of 3). As input alignment we used the same vertebrata alignment that we used in the case of Scorecons [50].

To determine if two normally distributed sets of data were significantly different from each other, or observed values were significantly different from a given mean, we performed two-sample and one-sample t-tests, respectively, using a statistical significance threshold of 0.05 to reject the null hypothesis.

For the Discrete Molecular Dynamics (DMD) simulations the following input sequences were generated: i) for the free MLL1 N-terminus, the amino acids 1–200 of MLL1 (UniProt: Q03164) were used to generate an extended structure in PyMol (The PyMOL Molecular Graphics System, Version 1.5.0.1 Schrödinger, LLC); ii) for menin and LEDGF/p75, sequences provided in PDB entry 3U88 were used, while for MLL1, the disordered regions removed from the construct were reinserted into the sequence and the purification tag was removed; iii) for the ternary complex supplemented with the disordered binding loop, PDB entry 2MSR was used. The structures were energy minimized by the DMD [51] protocol of Chiron (http://troll.med.unc.edu/chiron) [52]. Briefly, a short simulation (1,000 time unit/steps) using a high heat exchange factor (HEX  =  10) at a high temperature (0.7 temperature unit) was performed followed with a short simulation with a low heat exchange factor (HEX  =  0.1) at a low temperature (0.5 temperature unit). Cα and Cβ atoms were restrained. In all DMD simulations, including those combined with replica exchange, a united-atom representation is used to model proteins, in which all heavy atoms and polar hydrogen atoms of each amino acid are included [51, 53]. The solvent is implicitly modeled employing the Lazaridis-Karplus solvation model [54]. Long range electrostatic interactions are also implemented [51]. The πDMD software employed for simulations was kindly provided by Molecules in Action, LLC (http://www.moleculesinaction.com).

Replica exchange DMD (RX-DMD) simulations [53] were performed with 8 replicas at temperatures 0.5497, 0.5624, 0.5753, 0.5886, 0.6022, 0.6161, 0.6303, and 0.6448 temperature unit, for 4,000,000 time units. One frame (conformation) was generated every 200 time units. Anderson’s thermostat was used and the heat exchange factor was set to 0.1. At the end of a simulation, the frames from every trajectory were grouped by temperature for analysis. These simulations were run on the HPC of the Institute of Enzymology (RCNS, HAS, Hungary, supported by the Momentum Program of HAS).

Ψ and Φ torsion angles were determined by DSSP [55] for every structure at every temperature. The occurrence of torsion angles characteristic of α-helices was counted for every amino acid position and was divided by the total number of the structures (10,000). To see if the α-helical torsion angles arise at the level of individual amino acids or continuous helices are formed, the helical content for each frame was plotted along the amino acid sequence. All calculations and plotting were done in R [56].

## Reviewers’ comments

### Arne Elofsson (Stockholm University)

In the paper the authors highlights the obvious fact that proteins in histone lysine methylation are disordered. This fact is readily available to anyone through for instance uniprot annotations.

*Authors’ response: Thank you for your work and your suggestions that helped to improve the scientific quality of our manuscript. Our aim was to draw attention to the fact that although the information is indeed available, yet no, or very few experimental works are aimed at the study of these regions. In most papers published about SET domain proteins the regions outside the SET domain are completely neglected.*

The paper lacks statistical analysis.

*Authors’ response: We performed statistical analysis of the disorder tendency of the different histone modifying enzyme families and also the frequency of the predicted ELM motifs compared to a randomized sequence dataset. Additional statistical analyses are included in the revised version of the manuscript along with the statistics of the new results.*

Expressions such as “some, or many of the ELMs found in this study may participate in the interactions of HKMTs” needs to be statistically analysed. Is this an overrepresentation or not.

*Authors’ response: The original sentence begins with “Our suggestion is…”, which is meant to show that this was not, and without experimental proof, can not be a definitive statement. We have shown that the predicted ELMs are statistically overrepresented in HKMTs compared to the random expectation. We complemented our studies with a statistical analysis of ELMs that participate in protein-protein interactions and found that they occur at a higher frequency than in the randomized sequence set; the results are included in the revised manuscript. It needs to be emphasized though that this statistical enrichment needs experimental verification, as stated in the text.*

Also the paper is full of statements like “Recognizing the importance of protein disorder in the epigenetic regulation is crucial for a deeper understanding that may bring further development of this field.” which does not really provide any novel insights but is more off an argument.

*Authors’ response: The arguments are involved in the text to further highlight the need for experimental studies aimed at the regions of HKMTs aside from their globular domains. Since these regions are almost completely neglected in the structural and functional studies regarding these proteins, it is difficult to provide more than arguments at this point. However, we changed the wording to be less dramatic.*

It needs to at least be supported by a T-test showing that epigenetic regulation is more common in disordered regions than in ordered regions.

*Authors’ response: The involvement of disordered proteins/regions in chromatin remodelling and consequently the epigenetic regulation has been analyzed by Sandhu* [9] *who concluded that most chromatin-related proteins contain long intrinsically disordered region. Our statistical analysis showed that not all histone modifying enzyme families contain equal levels of intrinsic disorder and that histone lysine methyltransferases and histone acetyltransferases have significantly higher disorder level than the other histone modifying enzymes. In the lack of direct evidence of the enrichment of protein disorder in epigenetic regulation, more profound analysis can not be performed at the moment.*

Finally, the obvious fact that the proteins contain many Poly-Gln stretches (and many other interesting features) is not discussed at all. What is their role ? How is the charge important for binding?

*Authors’ response: We included an analysis of single amino acid repeat regions in the HKMTs in the manuscript texts. However, since no available studies aimed to uncover the role of these regions, we can only speculate about their possible functions, as is done now in the revised text.*

Finally the evolutionary analysis is very limited (not a single multiple sequence alignment or tree is presented).

*Authors’ response: Multiple sequence alignment was performed for the DISCONS analysis and ANCHOR site conservation analysis and the results are available at this link "*github.com/lazartomi/HKMT_2016_raw*". Because the detailed evolutionary analysis of the HKMTs was not an aim of our study and can be readily found in the literature and also because of the limitations of the Discovery Notes format, we did not see it necessary to present the alignment as a main figure.*

Therefore, I would suggest the authors to at the bare minimum do

1. a statistical analysis for all statements
  *Authors’ response: We have complemented the statistical analysis with analysing the frequency of ELMs participating in protein-protein interactions and the predicted ANCHOR sites and included the results in the manuscript text.*
2. provide an analysis of the sequences in more details
  *Authors’ response: We performed SEG analysis and the results are included and discussed in the manuscript. Further, we now speculate about the possible role of highly repetitive, low-complexity regions.*
3. provide a comparative genomics analysis
  *Authors’ response: We added an evolutionary analysis of the protein length and gene number of histone modifying enzyme families to the manuscript. Our results show that while the number of HKMTs does not differ significantly from most of the other histone modifying enzymes, they are generally longer in most of the evolutionary categories studied.*

### Piotr Zielenkiewicz (Institute of Biochemistry and Biophysics, Polish Academy of Sciences)

Based on a limited number of experimental facts, the Authors analyse the structural diversity of human histone lysine methyltransferases. This analysis leads them to a conclusion that disordered regions contain conserved binding sites and may play a role in epigenetic regulation. The paper is speculative, but based on solid knowledge and sound bioinformatics analysis. In my opinion the final hypothesis makes the manuscript worth publication.

I believe some recommendations to wet lab colleagues will add value to the MS and I believe the Authors can make such recommendations easily based on their analysis (and MD simulations?).

*Authors’ response: Thank you for your review and positive comments. We have included the suggested recommendations in the manuscript text.*

## Additional files

Additional file 1: Figure S1. Long intrinsic disorder in histone modifying protein families. (A) Number of amino acids in disordered regions longer than 80 aa. (B) Distribution of disordered region length. Dark gray: histone lysine methyltransferases, vertical dashes: histone arginine methyltransferases, light gray: histone acetyltransferases, dots: histone deacetylases, gray: histone demethylases, horizontal dashes: UniProt average. (C) Frequency of disordered and low complexity regions. Dark grey: low complexity regions, white: low complexity and disordered regions, horizontal dashes: disordered regions. Differences significant at 0.0001 ≤ p are marked with asterisk. (JPG 252 kb) Additional file 2: Figure S2. Protein length and number in histone modifying enzyme families. (A) Length of proteins in different histone modifying enzyme families (B) Number of proteins in histone modifying enzyme families. Dark gray: histone lysine methyltransferases, vertical dashes: histone arginine methyltransferases, light gray: histone acetyltransferases, dots: histone deacetylases, gray: histone demethylases. Differences compared to HKMTs that are significant at 0.0001 ≤ p are marked with asterisk. (PNG 108 kb) Additional file 3: Table S1. Sequence and disorder conservation of six human HKMTs and CBP and BRCA1 based on the multiple sequence alignment of vertebrate orthologs. (DOCX 55 kb) Additional file 4: Table S2. Human HKMTs with disorder rate (IUPred) higher than 50 %. Disorder %: the total proportion of amino acids with an IUPred score above 0.5. Longest IDR: the longest region with all amino acids having IUPred score above 0.5. Function: catalytic activities of the HKMTs. ELM motifs: Eukaryotic Linear Motifs collected from the ELM database. Experimentally verified motifs are typed with normal characters and predicted (e < 0.0001) with italic. Amino acid repeats: single amino acid repeat regions determined in the SEG analysis. (DOCX 95 kb) Additional file 5: Movie 1. Molecular dynamics simulation of the LEDGF/p75-menin interaction. Cyan: LEDGF/p75, green: menin (MOV 13166 kb) Additional file 6: Movie 2. Molecular dynamics simulation of the MLL1-LEDGF/p75-menin interaction based on PDB 3U88. Salmon: MLL1, cyan: LEDGF/p75, green: menin. Sidechains of MLL1 F148 and F151 are red. (MOV 7391 kb) Additional file 7: Movie 3. Molecular dynamics simulation of the MLL1-LEDGF/p75-menin interaction based on PDB 2MSR. Salmon: MLL1, cyan: LEDGF/p75, green: menin. Sidechains of MLL1 F148 and F151 are red. (MOV 12102 kb)

## Abbreviations

aa: Amino acid
CBP: CREB-binding protein
consANCHOR: Conserved ANCHOR
DOT: Disruptor of telomeric silencing
ELM: Eukaryotic linear motif
HAT: Histone acetyltransferase
HEX: Heat exchange factor
HKMT: Histone lysine methyltransferase
HRMT: Histone arginine methyltransferase
IDP: Intrinsically disordered protein
IDR: Intrinsically disordered region
LEDGF: Lens epithelium-derived growth factor
MLL: Mixed lineage leukemia
MSA: Multiple sequence alignment
NSD1: Nuclear receptor-binding SET Domain-containing protein 1
PTM: Posttranslational modifications
RX-DMD: Replica exchange - discrete molecular dynamics
SET: Suppressor of variegation 3–9, Enhancer of zeste and Trithorax
SNP: Single-nucleotide polymorphism
TAD: Transactivation domain
Ub: Ubiquitin

## References

[1] Copeland RA (2013). Molecular Pathways: Protein Methyltransferases in Cancer. Clin Cancer Res..

[2] Herz H-M, Garruss A, Shilatifard A (2013). SET for life: biochemical activities and biological functions of SET domain-containing proteins. Trends Biochem Sci..

[3] Feng Q, Qin F, Hengbin W, Ng HH, Hediye E-B, Paul T (2002). Methylation of H3-Lysine 79 Is Mediated by a New Family of HMTases without a SET Domain. Curr Biol..

[4] Rao RC, Dou Y (2015). Hijacked in cancer: the KMT2 (MLL) family of methyltransferases. Nat Rev Cancer..

[5] McGrath J, Trojer P (2015). Targeting histone lysine methylation in cancer. Pharmacol Ther..

[6] Tompa P. Structure and Function of Intrinsically Disordered Proteins. Boca Raton, FL, USA: CRC Press; 2009.

[7] Tantos A, Kalmar L, Tompa P (2015). The role of structural disorder in cell cycle regulation, related clinical proteomics, disease development and drug targeting. Expert Rev Proteomics..

[8] Wright PE, Dyson HJ (2015). Intrinsically disordered proteins in cellular signalling and regulation. Nat Rev Mol Cell Biol..

[9] Sandhu KS (2009). Intrinsic disorder explains diverse nuclear roles of chromatin remodeling proteins. J Mol Recognit..

[10] Schlessinger A, Avner S, Christian S, Esmeralda V, Markus S, Marco P (2011). Protein disorder—a breakthrough invention of evolution?. Curr Opin Struct Biol..

[11] Schad E, Tompa P, Hegyi H (2011). The relationship between proteome size, structural disorder and organism complexity. Genome Biol..

[12] Varadi M, Guharoy M, Zsolyomi F, Tompa P (2015). DisCons: a novel tool to quantify and classify evolutionary conservation of intrinsic protein disorder. BMC Bioinformatics..

[13] Chen JW, Pedro R, Uversky VN, Keith DA (2006). Conservation of Intrinsic Disorder in Protein Domains and Families: I. A Database of Conserved Predicted Disordered Regions. J Proteome Res.

[14] Capra JA, Singh M (2007). Predicting functionally important residues from sequence conservation. Bioinformatics..

[15] Ganguly D, Zhang W, Chen J (2012). Synergistic folding of two intrinsically disordered proteins: searching for conformational selection. Mol Biosyst..

[16] Wootton JC (1994). Non-globular domains in protein sequences: automated segmentation using complexity measures. Comput Chem..

[17] Coletta A, Alain C, Pinney JW, David S, James M, Pettifer SR (2010). Low-complexity regions within protein sequences have position-dependent roles. BMC Syst Biol..

[18] Weber JJ, Sowa AS, Binder T, Hübener J (2014). From pathways to targets: understanding the mechanisms behind polyglutamine disease. Biomed Res Int..

[19] Lupas A, Van Dyke M, Stock J (1991). Predicting coiled coils from protein sequences. Science..

[20] Schaefer MH, Wanker EE, Andrade-Navarro MA (2012). Evolution and function of CAG/polyglutamine repeats in protein-protein interaction networks. Nucleic Acids Res..

[21] Chiti F, Dobson CM (2006). Protein misfolding, functional amyloid, and human disease. Annu Rev Biochem..

[22] Dinkel H, Van Roey K, Michael S, Davey NE, Weatheritt RJ, Born D (2014). The eukaryotic linear motif resource ELM: 10 years and counting. Nucleic Acids Res..

[23] Zhang P, Lee H, Brunzelle JS, Couture J-F (2012). The plasticity of WDR5 peptide-binding cleft enables the binding of the SET1 family of histone methyltransferases. Nucleic Acids Res..

[24] Tesina P, Čermáková K, Hořejší M, Procházková K, Fábry M, Sharma S (2015). Multiple cellular proteins interact with LEDGF/p75 through a conserved unstructured consensus motif. Nat Commun..

[25] Dosztanyi Z, Meszaros B, Simon I (2009). ANCHOR: web server for predicting protein binding regions in disordered proteins. Bioinformatics..

[26] Kuntimaddi A, Achille NJ, Thorpe J, Lokken AA, Singh R, Hemenway CS (2015). Degree of recruitment of DOT1L to MLL-AF9 defines level of H3K79 Di- and tri-methylation on target genes and transformation potential. Cell Rep..

[27] Meyer C, Hofmann J, Burmeister T, Gröger D, Park TS, Emerenciano M (2013). The MLL recombinome of acute leukemias in 2013. Leukemia..

[28] McGinty RK, Kim J, Chatterjee C, Roeder RG, Muir TW (2008). Chemically ubiquitylated histone H2B stimulates hDot1L-mediated intranucleosomal methylation. Nature..

[29] Oh S, Jeong K, Kim H, Kwon CS, Lee D (2010). A lysine-rich region in Dot1p is crucial for direct interaction with H2B ubiquitylation and high level methylation of H3K79. Biochem Biophys Res Commun..

[30] Nielsen AL, Jørgensen P, Lerouge T, Cerviño M, Chambon P, Losson R (2004). Nizp1, a novel multitype zinc finger protein that interacts with the NSD1 histone lysine methyltransferase through a unique C2HR motif. Mol Cell Biol..

[31] Ernst P, Wang J, Huang M, Goodman RH, Korsmeyer SJ (2001). MLL and CREB bind cooperatively to the nuclear coactivator CREB-binding protein. Mol Cell Biol..

[32] De Guzman RN, Goto NK, Dyson HJ, Wright PE (2006). Structural basis for cooperative transcription factor binding to the CBP coactivator. J Mol Biol..

[33] Terranova R, Agherbi H, Boned A, Meresse S, Djabali M (2006). Histone and DNA methylation defects at Hox genes in mice expressing a SET domain-truncated form of Mll. Proc Natl Acad Sci U S A..

[34] de Boer J, Walf-Vorderwülbecke V, Williams O (2013). In focus: MLL-rearranged leukemia. Leukemia..

[35] Hegyi H, Buday L, Tompa P (2009). Intrinsic structural disorder confers cellular viability on oncogenic fusion proteins. PLoS Comput Biol..

[36] Kalmar L, Acs V, Silhavy D, Tompa P (2012). Long-range interactions in nonsense-mediated mRNA decay are mediated by intrinsically disordered protein regions. J Mol Biol..

[37] Tompa P (2014). Multisteric regulation by structural disorder in modular signaling proteins: an extension of the concept of allostery. Chem Rev..

[38] Arai M, Sugase K, Dyson HJ, Wright PE (2015). Conformational propensities of intrinsically disordered proteins influence the mechanism of binding and folding. Proc Natl Acad Sci U S A..

[39] Yokoyama A, Akihiko Y, Cleary ML (2008). Menin Critically Links MLL Proteins with LEDGF on Cancer-Associated Target Genes. Cancer Cell..

[40] Huang J, Gurung B, Wan B, Matkar S, Veniaminova NA, Wan K (2012). The same pocket in menin binds both MLL and JUND but has opposite effects on transcription. Nature..

[41] Cermáková K, Tesina P, Demeulemeester J, El Ashkar S, Méreau H, Schwaller J (2014). Validation and structural characterization of the LEDGF/p75-MLL interface as a new target for the treatment of MLL-dependent leukemia. Cancer Res..

[42] Murai MJ, Pollock J, He S, Miao H, Purohit T, Yokom A (2014). The same site on the integrase-binding domain of lens epithelium-derived growth factor is a therapeutic target for MLL leukemia and HIV. Blood..

[43] Goyal S, Gupta G, Qin H, Upadya MH, Tan YJ, Chow VTK (2012). VAPC, an human endogenous inhibitor for hepatitis C virus (HCV) infection, is intrinsically unstructured but forms a “fuzzy complex” with HCV NS5B. PLoS One..

[44] Dosztanyi Z, Csizmok V, Tompa P, Simon I (2005). IUPred: web server for the prediction of intrinsically unstructured regions of proteins based on estimated energy content. Bioinformatics..

[45] Potenza E, Di Domenico T, Walsh I, Tosatto SCE (2015). MobiDB 2.0: an improved database of intrinsically disordered and mobile proteins. Nucleic Acids Res.

[46] Oates ME, Romero P, Ishida T, Ghalwash M, Mizianty MJ, Xue B (2013). D^2^P^2^: database of disordered protein predictions. Nucleic Acids Res..

[47] Stark C, Breitkreutz B-J, Reguly T, Boucher L, Breitkreutz A, Tyers M (2006). BioGRID: a general repository for interaction datasets. Nucleic Acids Res..

[48] Wu T-J, Shamsaddini A, Pan Y, Smith K, Crichton DJ, Simonyan V (2014). A framework for organizing cancer-related variations from existing databases, publications and NGS data using a High-performance Integrated Virtual Environment (HIVE). Database.

[49] Forbes SA, Beare D, Gunasekaran P, Leung K, Bindal N, Boutselakis H (2015). COSMIC: exploring the world’s knowledge of somatic mutations in human cancer. Nucleic Acids Res..

[50] Valdar WSJ (2002). Scoring residue conservation. Proteins: Struct Funct Genet.

[51] Shirvanyants D, Ding F, Tsao D, Ramachandran S, Dokholyan NV (2012). Discrete molecular dynamics: an efficient and versatile simulation method for fine protein characterization. J Phys Chem B..

[52] Ramachandran S, Kota P, Ding F, Dokholyan NV (2011). Automated minimization of steric clashes in protein structures. Proteins..

[53] Ding F, Tsao D, Nie H, Dokholyan NV (2008). Ab initio folding of proteins with all-atom discrete molecular dynamics. Structure..

[54] Lazaridis T, Karplus M (1999). Effective energy function for proteins in solution. Proteins..

[55] Kabsch W, Sander C (1983). Dictionary of protein secondary structure: pattern recognition of hydrogen-bonded and geometrical features. Biopolymers..

[56] R Core Team. R: A Language and Environment for Statistical Computing. R Foundation for Statistical Computing; 2015; Available: https://www.R-project.org

