# Supplementary figures and images for: Intrinsic protein disorder in histone lysine methylation
Source: Biol Direct. 2016 Jun 30;11:30. doi: 10.1186/s13062-016-0129-2 (PMC4928265; doi:10.1186/s13062-016-0129-2)

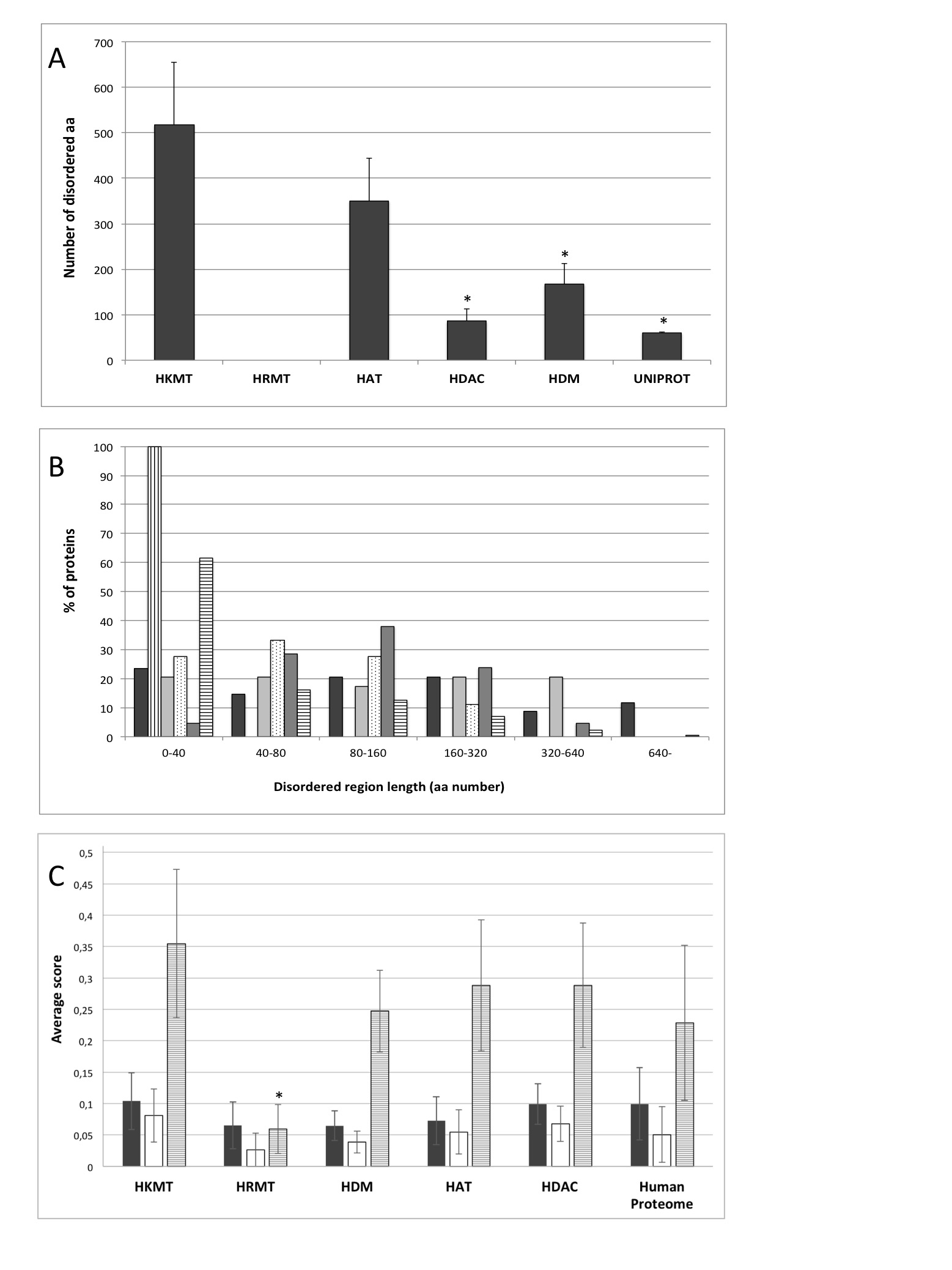

Supplement: Additional file 1: Figure S1. — Long intrinsic disorder in histone modifying protein families. (A) Number of amino acids in disordered regions longer than 80 aa. (B) Distribution of disordered region length. Dark gray: histone lysine methyltransferases, vertical dashes: histone arginine methyltransferases, light gray: histone acetyltransferases, dots: histone deacetylases, gray: histone demethylases, horizontal dashes: UniProt average. (C) Frequency of disordered and low complexity regions. Dark grey: low complexity regions, white: low complexity and disordered regions, horizontal dashes: disordered regions. Differences significant at 0.0001 ≤ p are marked with asterisk. (JPG 252 kb) [file 13062_2016_129_MOESM1_ESM.jpg]

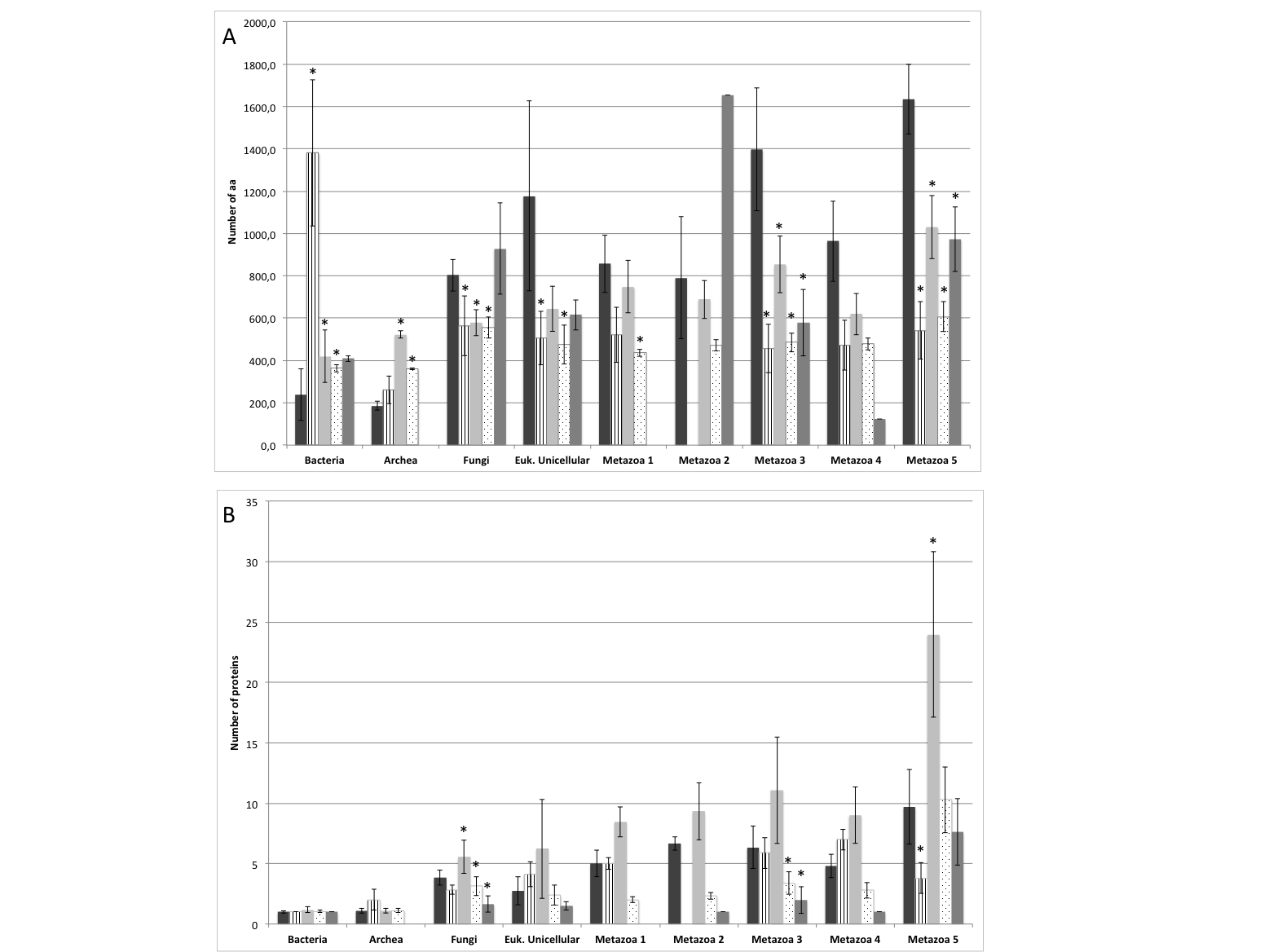

Supplement: Additional file 2: Figure S2. — Protein length and number in histone modifying enzyme families. (A) Length of proteins in different histone modifying enzyme families (B) Number of proteins in histone modifying enzyme families. Dark gray: histone lysine methyltransferases, vertical dashes: histone arginine methyltransferases, light gray: histone acetyltransferases, dots: histone deacetylases, gray: histone demethylases. Differences compared to HKMTs that are significant at 0.0001 ≤ p are marked with asterisk. (PNG 108 kb) [file 13062_2016_129_MOESM2_ESM.png]
