# Supplementary material for: Intrinsic protein disorder in histone lysine methylation
Source: Biol Direct. 2016 Jun 30;11:30. doi: 10.1186/s13062-016-0129-2 (PMC4928265; doi:10.1186/s13062-016-0129-2)
Supplement: Additional file 3: Table S1. — Sequence and disorder conservation of six human HKMTs and CBP and BRCA1 based on the multiple sequence alignment of vertebrate orthologs. (DOCX 55 kb) [file 13062_2016_129_MOESM3_ESM.docx]

| Name | Length | #species in the MSA | %constrained residues | %flexible residues | %non-conserved residues |
| --- | --- | --- | --- | --- | --- |
| MLL4 | 5537 | 23 | 96% | 0% | 4% |
| MLL1 | 3969 | 24 | 95% | 1% | 5% |
| SUV420H1 | 885 | 22 | 94% | 0% | 6% |
| NSD1 | 2696 | 23 | 87% | 0% | 13% |
| PRDM2 | 1718 | 23 | 88% | 0% | 12% |
| DOT1L | 1739 | 22 | 82% | 0% | 18% |
| CBP | 2442 | 22 | 97% | 1% | 2% |
| BRCA1 | 1863 | 24 | **67%** | **17%** | **18%** |

**Additional file 3: Table S1.** Sequence and disorder conservation of six human HKMTs and CBP and BRCA1 based on the multiple sequence alignment of vertebrate orthologs.
