# Supplementary material for: Intrinsic protein disorder in histone lysine methylation
Source: Biol Direct. 2016 Jun 30;11:30. doi: 10.1186/s13062-016-0129-2 (PMC4928265; doi:10.1186/s13062-016-0129-2)
Supplement: Additional file 4: Table S2. — Human HKMTs with disorder rate (IUPred) higher than 50 %. Disorder %: the total proportion of amino acids with an IUPred score above 0.5. Longest IDR: the longest region with all amino acids having IUPred score above 0.5. Function: catalytic activities of the HKMTs. ELM motifs: Eukaryotic Linear Motifs collected from the ELM database. Experimentally verified motifs are typed with normal characters and predicted (e < 0.0001) with italic. Amino acid repeats: single amino acid repeat regions determined in the SEG analysis. (DOCX 95 kb) [file 13062_2016_129_MOESM4_ESM.docx]

Additional file 4: Table S2

| Name | UniProt Acc | %disorder^IUPRED^ | Longest IDR (aa) | Function | ELM motifs | Amino acid repeats |
| --- | --- | --- | --- | --- | --- | --- |
| MLL5 | Q8IZD2 | 78.6% | 720 | H3K4me1/2 | *DEG_ODPH_VHL_1 (63-66), DEG_Kelch_Keap1_1 (1202-1207)* | P1544-1552; P1627-1634; P1678-1684; P1715-1723 |
| MLL4 | O14686 | 74.5% | 1354 | H3K4me1 | LIG_WD40_WDR5_WIN_1 (5338-5344), LIG_KEPE_1 (4415-4421), *DOC_PIKK_1 (3023-3031, 3034-3041), DOC_MAPK_2 (2536-2539), LIG_RRM_PRI_1 (4255-4262), LIG_EH1_1 (5177-5185), DEG_Kelch_Keap1_1 (2598-2603), DEG_SIAH_1 (1187-1195), LIG_MYND_2 (1196-1200)* | Q2811-2816; Q3274-3282; Q3599-3612; Q3679-3684; Q3735-3745; Q3754-3759; Q3856-3863; Q3913-3919; Q3932-3939; Q3941-3947; Q3949-3954; Q3956-3965; Q3967-3974; Q3988-3994 |
| MLL1 | Q03164 | 71.8% | 479 | H3K4me1 | LIG_WD40_WDR5_WIN_1 (3763-3769), CLV_TASPASE1 (2664-2670, 2716-2722), *LIG_HCF-1_HBM_1 (1799-1802), DEG_ODPH_VHL_1 (3243-3254), DOC_MAPK_2 (796-799)* | G17-23; A61-67; S90-98 |
| SET1A | O15047 | 70.4% | 600 | H3K4me1 | LIG_WD40_WDR5_WIN_1 (1493-1499) *LIG_HCF-1_HBM_1* (1299-1302) | S345-357; G441-448; P604-616; P647-654; S1035-1058; E1370-1375; P1403-1412 |
| SET1B (SETD1B) | Q9UPS6 | 69.1% | 511 | H3K4me1 | LIG_WD40_WDR5_WIN_1 (1746-1752), *LIG_HCF-1_HBM_1 (326-329), LIG_EF_ALG2_ABM_2 (389-393, 682-687, 699-703), DEG_ODPH_VHL_1 (1218-1230)* | P691-699; P710-718; A799-805; E1081-1087; D1105-1110; E1185-1194; P1590-1598 |
| SETD5 | Q9C0A6 | 66.9% | 447 | Unknown | - | - |
| MLL3 | Q8NEZ4 | 66.4% | 1405 | H3K4me1 | LIG_WD40_WDR5_WIN_1 (4708-4714), *LIG_Rb_pABgroove_1 (237-245), LIG_Sin3_3 (1524-1531), DOC_MAPK_2 (2482-2485), DOC_PIKK_1 (2713-2720, 2741-2748), LIG_NBox_RRM (3553-3563), LIG_KEPE_1 (4024-4030), LIG_EH1_1 (4547-4555)* | P14-19; Q1780-1787 |
| MLL2 | Q9UMN6 | 65.6% | 783 | H3K4me1 | LIG_WD40_WDR5_WIN_1 (2509-2515), *LIG_MYND3 (769-773), LIG_HCF-1_HBM_1 (1537-1540), LIG_WRPW_2 (1783-1786), LIG_RRM_PRI_1 (1996-2003, 2238-2245), CLV_TASPASE1 (2060-2066)* | P430-435; P2253-2259 |
| PRDM2 (RIZ) | Q13029 | 64.3% | 227 | H3K9me1 | *LIG_EH1_1 (595-603), LIG_MYND_2 (962-966)* | E268-276; S1052-1063; S1065-1072 |
| SET2 (SETD2) | Q9BYW2 | 61.2% | 518 | H3K36me3 | - | P190-196 |
| SUV420H1 | Q4FZB7 | 61.0% | 323 | H4K20me3 | - | - |
| NSD1 | Q96L73 | 57.6% | 421 | H3K36me1, H4K20me1 | *LIG_KEPE_2 (1338-1346), LIG_MYND_3 (2483-2487)* | - |
| DOT1L | Q8TEK3 | 56.4% | 466 | H3K79me1/2/3 | *DEG_ODPH_VHL_1 (932-945)* | P1592-1602 |
| PRDM10 | Q9NQV6 | 54.4% | 270 | Unknown | - | Q1019-1026 |
| PRDM8 | Q9NQV8 | 50.4% | 324 | H3K9me2 | *DEG_Kelch_Keap1_1 (375-380)* | G203-209; Q212-217; A587-598 |
| SETD8 (SET8) | Q9NQR1 | 50.4% | 146 | H4K20me1 | DEG_CRL4_CDT2_1 (220-231), *DEG_PIKK_1 (242-250)* | A12-23 |
